# Kinetically Limited Growth of Dendritic Tin Oxide Thin Films: a Machine Learning Study beyond the Structure Zone Diagram

**DOI:** 10.1002/advs.202504627

**Published:** 2025-05-29

**Authors:** Denis Music, Xuelian Xiao, Rami Naser, Keke Chang, Grzegorz Sadowski, Pär A. T. Olsson

**Affiliations:** ^1^ Department of Materials Science and Applied Mathematics Malmö University Malmö SE‐205 06 Sweden; ^2^ Biofilms Research Center for Biointerfaces Malmö University Malmö SE‐205 06 Sweden; ^3^ State Key Laboratory of Advanced Marine Materials Ningbo Institute of Materials Technology and Engineering Chinese Academy of Sciences Ningbo 315201 China; ^4^ Division of Mechanics Materials and Component Design Lund University P.O. Box 118 Lund SE‐221 00 Sweden

**Keywords:** density functional theory, machine learning, nanomaterials, thin films, tin oxides

## Abstract

Even after fifty years since its introduction, the empirical Thornton's structure zone diagram remains a valuable tool for predicting thin film microstructure. This diagram is essential for understanding the correlation between synthesis, composition, structure, and physical properties in emerging applications. In this work, we critically appraise this diagram by examining Sn─O thin films grown at room temperature using reactive magnetron sputtering. Based on transmission electron microscopy, Sn_0.6_O_0.4_ thin films form dendrites featuring nanosized Sn and SnO grains, rather than columns, which are not captured by the structure zone diagram. Using density functional theory and machine learning, we constructed a model to explain this unusual microstructure on the atomic scale. Kinetically limited surface diffusion yields SnO islands on Sn(001), which constitute the initial stage of dendrite formation. This study provides the potential to devise models for thin film microstructure evolution, enhancing performance in advanced applications, such as green energy generation and storage.

## Graphical abstract

This work presents a critical evaluation of the empirical Thornton's structure zone diagram, a cornerstone in predicting thin film microstructure. Through transmission electron microscopy, we observe that Sn─O thin films form nanosized dendrites, which are not expected according to the structure zone diagram. We employ density functional theory and machine learning to construct a new model that elucidates the underlying atomic‐scale mechanisms.

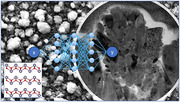

## 1 Introduction

Thin films are nowadays integrated into almost all advanced technologies, yet many scientific questions remain open about the fundamental atomic processes that drive their nucleation and growth, often affecting their efficiency and lifespan. They frequently serve as a validation (or realization) platform for various physical concepts, as exemplified by Majorana particles,^[^ ^1^ ^]^ signifying their importance in fundamental research. Thornton's structure zone diagram is a half‐a‐century‐old framework that is still frequently used to predict the microstructure evolution of thin films synthesized by evaporation, magnetron sputtering, and other non‐equilibrium plasma‐based techniques.^[^ ^2^ ^]^ It is an empirical diagram derived for metals,^[^ ^2^ ^]^ which tends to be used for more complex systems (multiple elements).^[^ ^3^, ^4^, ^5^ ^]^ The applicability of the structure zone diagram beyond simple metals has been criticized in literature, e.g. for the growth of amorphous thin films.^[^ ^6^ ^]^ Thin films synthesized from the vapor phase are typically governed by atomic processes, where atoms (or ions) arrive on a substrate, diffuse, nucleate, and form clusters, which upon coalescence give rise to microstructure.^[^ ^7^ ^]^ The structure zone diagram in its most accepted form encompasses physical parameters that are anchored in thermodynamics (melting point) and kinetics (diffusion).^[^ ^8^, ^9^ ^]^ It classifies the microstructure of thin films into distinct zones, each characterized by different growth mechanisms and hence surface morphologies.^[^ ^2^ ^]^ At low homologous temperature (substrate temperature divided by melting point) and low kinetic energy of incoming ions from the plasma (typically less than ≈20–30 eV, controlled by pressure or substrate bias in the case of sputtering), thin films tend to be amorphous (or highly porous) due to limited diffusion.^[^ ^2^ ^]^ At the homologous temperature higher than ≈0.3 and low kinetic energy, surface, and grain boundary diffusion is activated, typically yielding a well‐defined columnar microstructure with relatively dense boundaries.^[^ ^2^ ^]^ This is the most common appearance of polycrystalline thin films. At the homologous temperature above 0.5 and high kinetic energy (higher than ≈40–50 eV), bulk diffusion dominates, which leads to the formation of large, equiaxed grains.^[^ ^2^ ^]^ Even though the zone boundaries and the number of zones are still under dispute, this straightforward diagram has often been utilized in the thin film physics community. Over the years, there have been modifications to the original approach. For instance, to include cathodic arc synthesis, the range of kinetic energy was extended.^[^ ^10^ ^]^ Furthermore, the angle of the deposition flux was added to account for glancing angle deposition.^[^ ^11^ ^]^ Some approaches for modifying the structure zone diagram explicitly include shadowing effects to delineate open microstructures.^[^ ^12^ ^]^ Furthermore, there are several Monte Carlo simulations carried out, supporting the empirical structure zone diagram.^[^ ^13^, ^14^, ^15^ ^]^ However, a lot of physics is still left out, such as surface and defect formation energy. Any successful nucleation model should contain surface energy data, which implies that any description of microstructure evolution should also include it, which is currently not the case for the structure zone diagram.

Tin oxides (Sn─O) are selected herein to critically appraise the performance of the structure zone diagram even though it was not proposed for oxides. These oxides, mainly tin(II) stannous oxide (SnO) and tin(IV) stannic oxide (SnO₂), are important systems known for their unique physical and chemical properties as well as wide‐ranging functional applications. SnO typically forms a tetragonal (litharge, P4/nmm) or monoclinic layered structure (massicot, P1 or alt. orthorhombic Pbcn) and acts as a p‐type semiconductor (bandgap in the range of 2–3 eV).^[^ ^16^, ^17^, ^18^ ^]^ SnO_2_ is the most stable form based on Gibbs free energy and likely the most explored tin oxide, commonly adopting a tetragonal rutile structure (P4_2_/mnm).^[^ ^19^, ^20^, ^21^ ^]^ SnO₂ is an n‐type semiconductor with a wide bandgap of about 3.6 eV, which makes it suitable for various high‐tech electronic applications such as transparent conductive oxide in solar cells, various sensors, flat‐panel displays, touch screens, light‐emitting diodes, photodetectors, and electrodes in lithium‐ion batteries,^[^ ^19^, ^20^, ^21^ ^]^ to name a few. In general, tin oxides are known for their adjustable electrical properties through doping or morphological control.^[^ ^22^ ^]^ Hence, the predictability of tin oxide morphology is essential for property‐performance correlations.

The least explored region in the Sn─O binary phase diagram is located below 50 at.% of O, where mixtures of Sn and SnO are expected at equilibrium conditions.^[^ ^23^ ^]^ Both SnO and SnO_2_ are promising thermoelectric phases since the Seebeck coefficient is in the range of 400 and −250 µV K^−1^ at room temperature, respectively,^[^ ^24^, ^25^ ^]^ but they suffer from insufficient electrical conductivity in their pristine states. This is where mixtures of Sn and SnO or even Sn, SnO, and SnO_2_ could be beneficial. Furthermore, core‐shell Sn/Sn─O nanostructures are also of interest^[^ ^26^ ^]^ as they exhibit a similar phase combination. Applications of SnO are emerging, whereby catalysis and sensors have recently been considered.^[^ ^27^, ^28^ ^]^ Hence, the Sn‐rich region in the Sn─O binary phase diagram should be explored, whereby the structure and morphology evolution are of key importance for designing novel devices and unraveling the underlying growth mechanisms.

**Figure 1:**
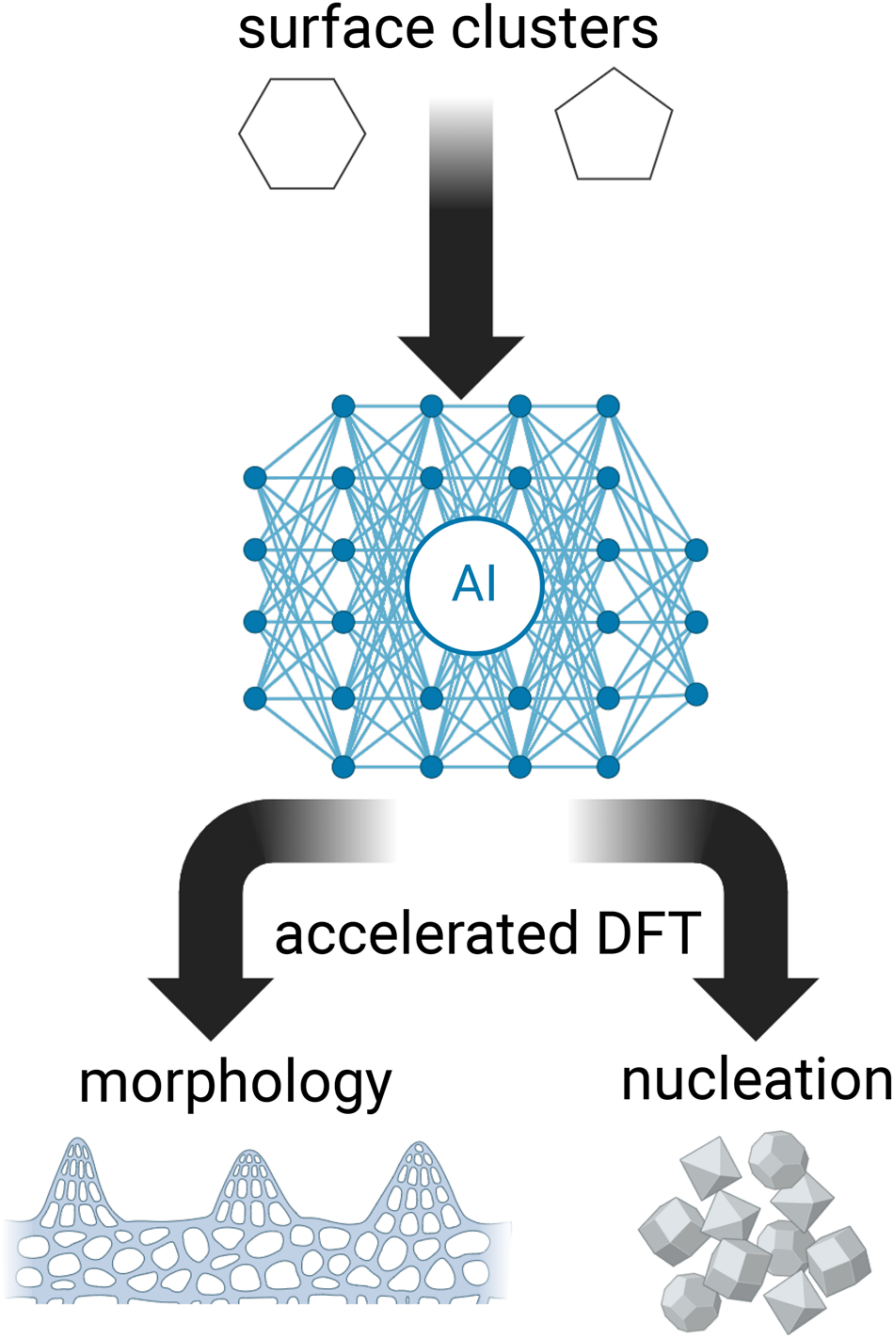
Generic workflow chart for microstructure evolution proposed in the current study. Surface clusters are generated by DFT serving as input to AI, which allows for acceleration of DFT to vindicate surface morphology and nucleation.

In this work, the applicability of the structure zone diagram is critically appraised on Sn‐rich tin oxide thin films synthesized by reactive magnetron sputtering at room temperature aiming at explaining the growth mechanisms. The structure zone diagram was originally derived for metals,^[^ ^2^ ^]^ but here we assess it on oxide thin films. Experimental and theoretical methods are synergistically employed. Using scanning electron microscopy (SEM) and transmission electron microscopy (TEM), we show that dendritic microstructure emerges, which is not captured by the structure zone diagram that predicts columnar samples. A correlative theoretical model based on density functional theory (DFT) and artificial intelligence (AI, machine learning) is constructed to explain microstructure evolution. The generic workflow chart is presented in **Figure** **1**. Surface clusters on SnO with up to seven atoms (both Sn and O) are generated by DFT and serve as input to machine learning. This allows for acceleration of DFT to describe surface morphology and nucleation of Sn─O samples grown experimentally, thus replacing the structure zone diagram in this specific case. In the order of 10^7^ computational steps can be saved using this strategy.

## 2 Results

### 2.1 Experimental Evidence for the Limitation of the Structure Zone for Tin Oxide

When a thin film sample exhibits a Sn‐rich composition (Sn >50 at.%), the following prediction can be obtained from the structure zone diagram.^[^ ^2^ ^]^ According to the equilibrium phase diagram established for bulk Sn─O, the melting points of the Sn and SnO phases are ≈230 and 1080 °C, respectively.^[^ ^29^ ^]^ Based on the structure zone diagram,^[^ ^2^ ^]^ it takes ≈30% of the melting point to trigger surface diffusion and likely form crystalline samples. It should also be noted that some adatom mobility is induced through ion bombardment, present in sputtering discharges which may further lower the growth temperature requirement. Hence, Sn may be crystalline at room temperature, while SnO is expected to be amorphous. Morphologically, columnar features are expected to arise. It is also possible to speculate about phase constitution based on the phase diagram constructed for Sn─O nanoparticles,^[^ ^23^ ^]^ implying that both Sn and SnO could be grown crystalline at room temperature. The surface energy of SnO basal planes (SnO(001))^[^ ^30^ ^]^ was reported to be as low as *σ* = 0.08 J m^−2^. It is known^[^ ^31^ ^]^ that the energy barrier for homogeneous nucleation scales with *σ* ^3^. A low barrier implies that it is energetically favorable to nucleate a phase. It appears that SnO may nucleate at even lower temperatures than predicted by the structure zone diagram. Hence, it can be anticipated that Sn and SnO form crystalline features with a columnar microstructure.

**Figure 2:**
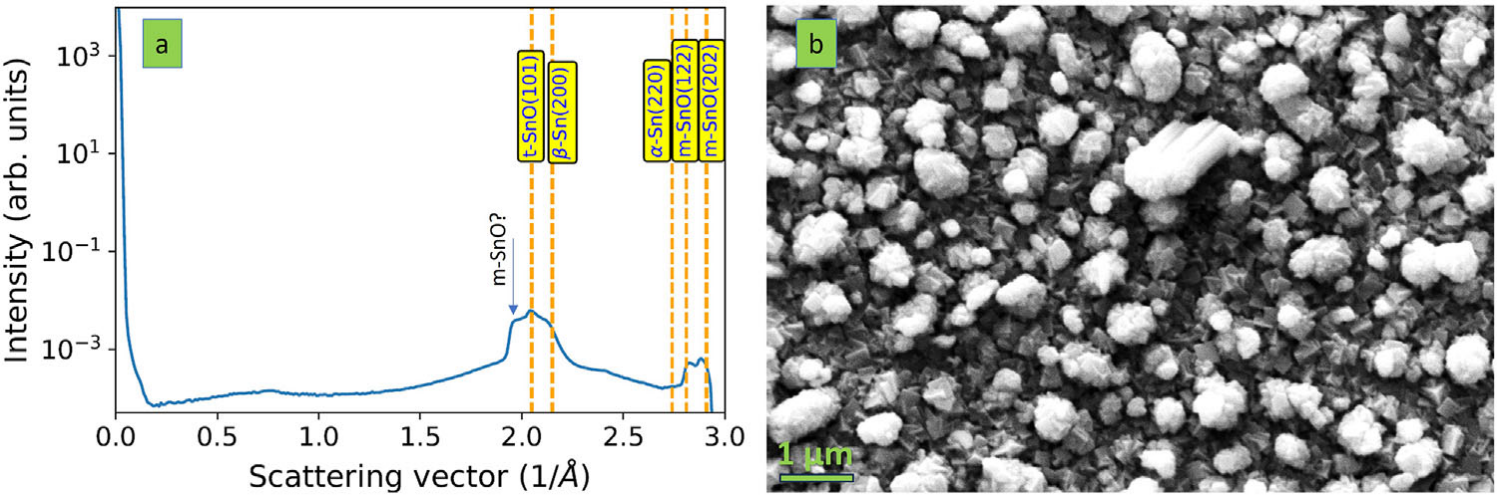
Structure and morphology of Sn_0.6_O_0.4_. a) WAXS data on Sn_0.6_O_0.4_ thin film, implying that α‐Sn (Fd‐3m), β‐Sn (I4/mmm), t‐SnO (P4/nmm), and m‐SnO (P1) crystallites form. The dashed lines indicate the expected position of Bragg's peaks. The arrow indicates the peak with the highest uncertainty. b) SEM image of Sn_0.6_O_0.4_ thin film showing a rough surface with dendritic features.

Using reactive magnetron sputtering and subsequently energy dispersive x‐ray analysis (EDX), the composition of a grown thin film was determined to be: 60 ± 1 at.% Sn and 40 ± 1 at.% O. Henceforth, this composition is referred to as Sn_0.6_O_0.4_. No impurities were detected. This EDX measurement was carried out in conjunction with SEM. Using TEM and the corresponding compositional analysis, more details can be uncovered (see Figures S1 and S2, Supporting Information). Some variations in the composition over the film thickness occur, whereby higher O contents are present close to the sample surface. This composition meets the phase diagram requirement to form a mixture of Sn and SnO phases, which was probed by wide‐angle x‐ray scattering (WAXS) and selected area electron diffraction (SAED). The WAXS data are presented in **Figure** **2**A. The peaks in the WAXS diagram are broad, indicating a short coherency length (small grains). The sample likely contains Sn (Fd‐3m and I4_1_/amd) and SnO (P4/nmm and P1) crystallites. While it is expected to form crystalline Sn based on the structure zone diagram, crystalline SnO is not anticipated. Our hypothesis that SnO can nucleate even at room temperature seems justified. The small feature size revealed by WAXS is consistent with the SEM image provided in Figure 2B. It reveals a rough surface with open features that are clearly distinguishable. These features vary in size, indicating a heterogeneous distribution across the Sn_0.6_O_0.4_ surface. The morphology is notably dendritic, characterized by tree‐like branching structures. This dendritic pattern suggests a growth process where branches extend outward, creating a complex, interconnected Sn─O network. The varying sizes of the Sn─O features contribute to a coarse and intricate surface morphology. This shows the limitation of the structure zone diagram for the present application since columnar morphology is expected. Hence, the structure zone diagram does not describe the growth of the Sn_0.6_O_0.4_ thin film.

**Figure 3:**
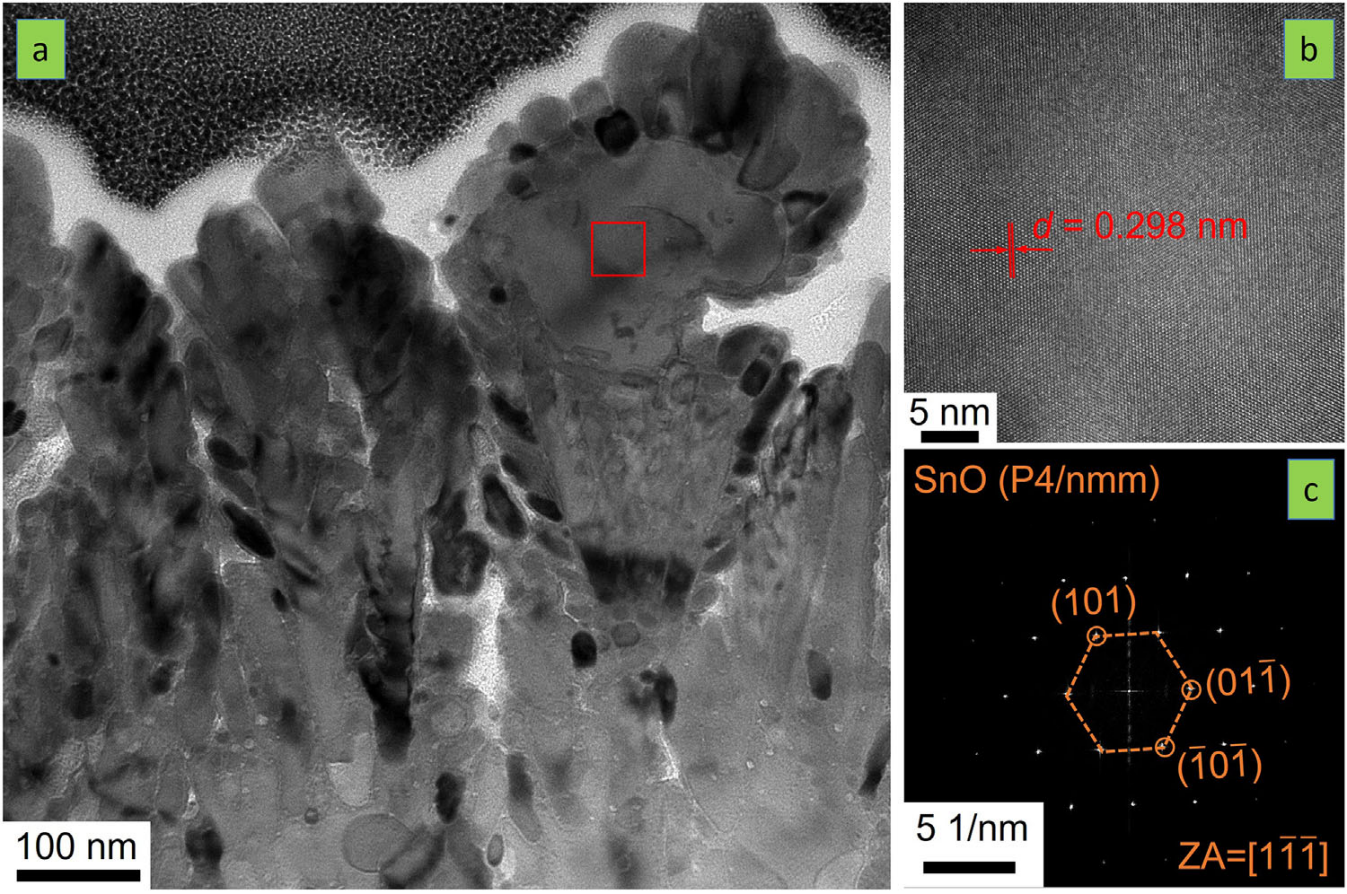
TEM analysis of Sn_0.6_O_0.4_. a) Bright‐field image of the sample analyzed. The area marked by the square is where the high‐resolution TEM analysis was undertaken. b) High‐resolution TEM of a grain exhibiting SnO structure (P4/nmm). The interplanar spacing (*d*) is displayed. The corresponding SEAD pattern is provided in image c), where the zone axis (ZA) is also indicated.

A more detailed structural and morphological analysis of Sn_0.6_O_0.4_ was provided using TEM (see **Figures** **3** and S3, Supporting Information). The aforementioned dendritic morphology suggested by SEM (Figure 2B) is confirmed (Figure 3A and Figure S3A, Supporting Information). Close to the substrate (bottom of Figure 3A and Figure S3A, Supporting Information), the sample is denser without visible voids. As the thickness increases, open features evolve. There are small dendritic features in the range of 100 nm, but others seem to extend over 300 nm or more. Each dendrite appears to contain even smaller structures. When analyzed at a higher resolution (Figure 3B and Figure S3B, Supporting Information), small grains in the range of 15 to 20 nm can be distinguished. These small grains readily overlap, which carries uncertainty in the SAED investigation and does not reinforce the analysis of interfaces. Nevertheless, SnO (P4/nmm in Figure 3C) and Sn (Fd‐3m in Figure S3C, Supporting Information) seem to be present. This is consistent with the WAXS data in Figure 2A. Hence, based on both SEM and TEM data, Sn_0.6_O_0.4_ exhibits a mixture of nm‐sized SnO and Sn crystalline grains with a dendritic microstructure. This dendritic growth is not captured by the structure zone diagram, which needs to be clarified.

### 2.2 Machine Learning Model

**Figure 4:**
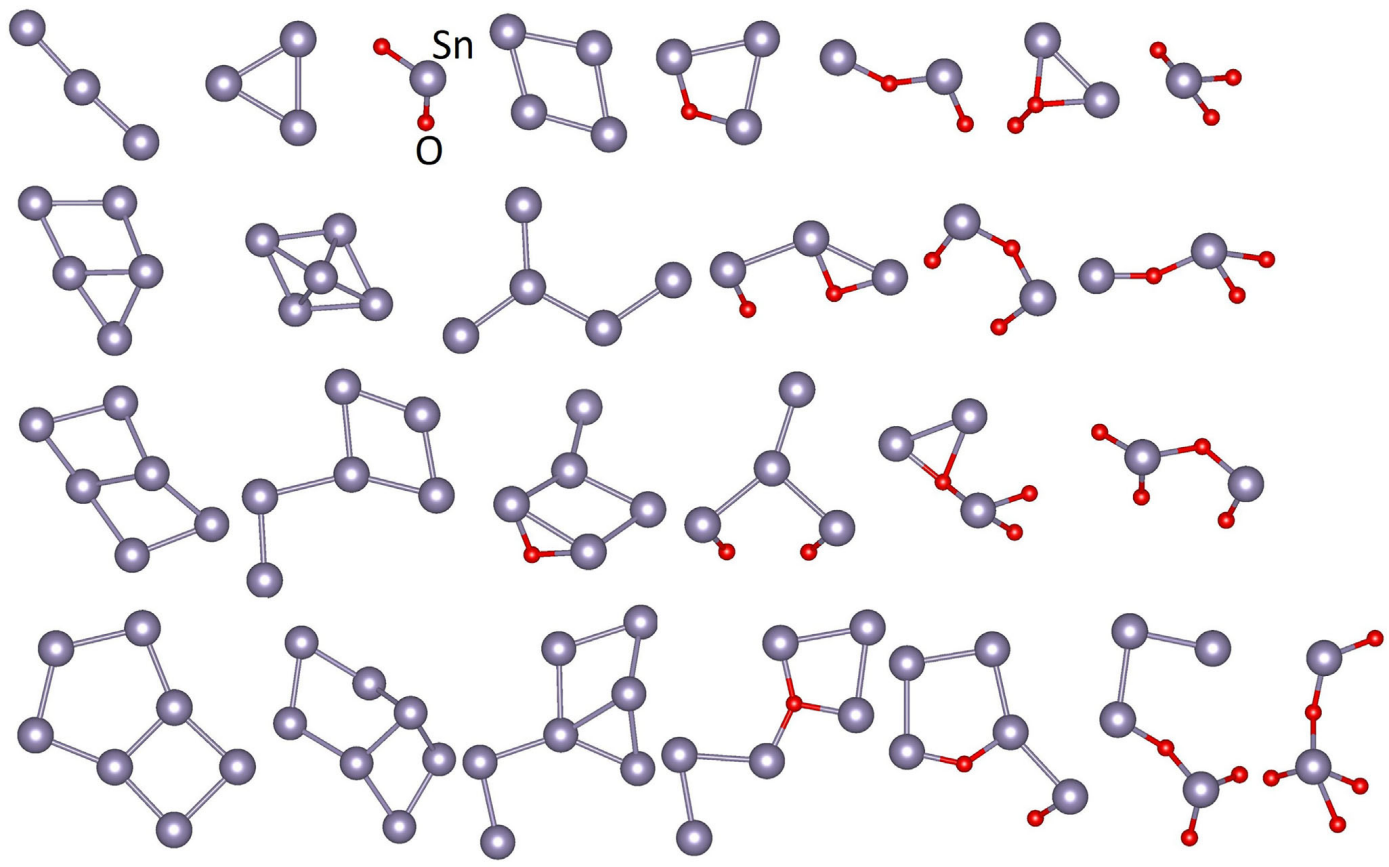
Sn─O cluster pretotypes larger than binary units. These 27 cluster prototypes (as well as monomers and dimers) were placed on SnO(001). In total, 93 clusters were considered. Adsorption energies at 0 K were calculated by DFT, serving as input for machine learning.

Following the workflow outlined in Figure 1, a DFT dataset pertaining to the adsorption of Sn─O clusters on Sn0(001) was used to derive a machine‐learning model based on an artificial neural network. These clusters form on a substrate as a result of adatom mobility, but here we accelerate the atomic processes by considering clusters. Placing them 2 Å above the surface, which is an arbitrary value (a common bond length), provides reproducibility. The prototypes of such surface clusters are provided in **Figure** **4**. Ranging from isolated adatoms to Sn─O clusters with 7 atoms (heptamers), different structural prototypes were obtained on the Sn‐terminated SnO(001) surface shown in Figure S4 (Supporting Information), and the adsorption energy values were extracted at 0 K (see Supporting Information for the whole DFT dataset). In total, 93 clusters were taken into account, built from the 27 structural prototypes shown in Figure 4, which is a considerable amount for DFT modeling. Some clusters are highly symmetric, linear, or nearly rectangular, but some have highly irregular shapes (see Figure 4), which is induced by the surface sites on SnO(001). It is plausible that this prototype diversity allows for satisfactory statistics.

**Figure 5:**
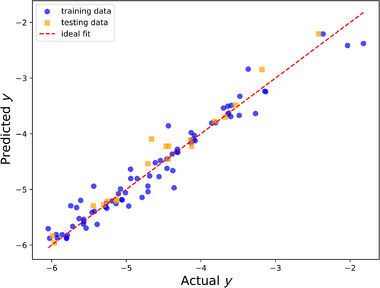
Artificial neural network model with 93 data points. The parameter *y* is the adsorption energy in eV/atom. The prediction of the artificial neural network was evaluated, obtaining an accuracy of 95.7% based on *R* ^2^, which is the coefficient of determination. The input was randomly split into training (80%) and testing (20%) datasets.

The DFT dataset was assessed and scaled, and the following features (descriptors) were considered: i) the average bond length within a cluster and its neighboring surface atoms, ii) the number of the nearest neighbors with the SnO surface (P4/nmm), iii) the number of the nearest neighbors within a cluster, and iv) the average electronegativity^[^ ^32^ ^]^ (between a cluster and the SnO surface). These features were selected to enable a possible utility of this model for experimentalists since DFT‐specific features (e.g., the density of states at the Fermi level or Bader charge) cannot be a priori known. The accuracy of the model is of course the key parameter to judge this selection. Furthermore, including the average bond length between a cluster and a surface, rather than only within the cluster, is motivated by the ambition to describe the microstructural evolution and not the formation of isolated clusters in a vacuum. These clusters are then incorporated into a growing thin film. The DFT dataset is plotted against these features in Figure S5 (Supporting Information). It appears that the calculated adsorption energy correlates well with the average bond length within a cluster and its neighboring surface atoms, but substantial scattering occurs. Conversely, other features are less explicit. Using an artificial neural network with two hidden layers, one with 64 neurons and the other with 32 neurons, and a constant learning rate of 0.001, an accuracy of 95.7% was achieved (see **Figure** **5**), which is satisfactory. It is unlikely that overfitting occurred since the model is simple and regularization was used. Hence, the adsorption of Sn─O clusters on relevant surfaces can be predicted, which is an essential prerequisite for explaining the microstructure obtained experimentally (Figure 3).

### 2.3 Evolution of Tin Oxide Microstructure

**Figure 6:**
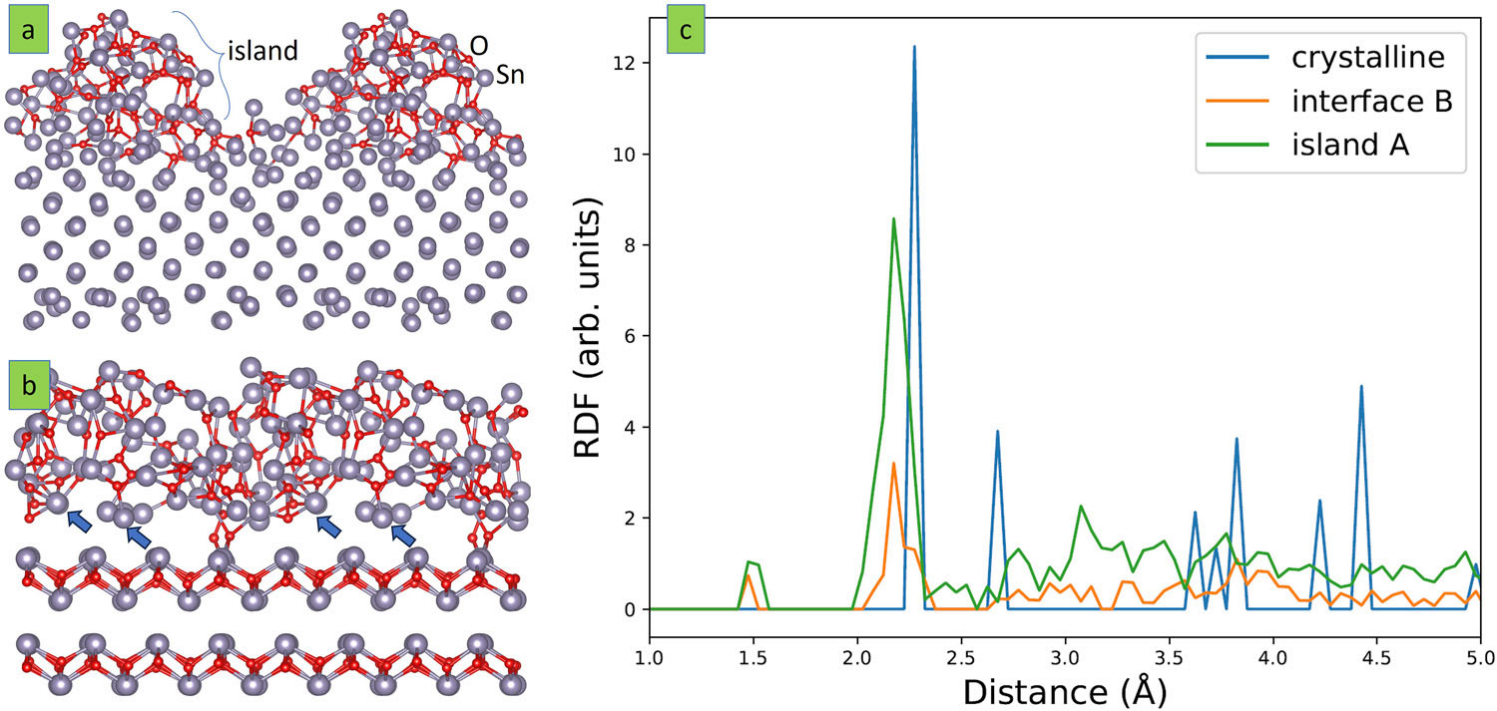
Evolution of Sn─O microstructure. a) SnO island formed on a Sn(001) slab. b) SnO island (taken from a)) on a SnO(001) slab. The blue arrows indicate the observed ordering. Both images a) and b) are doubled laterally to enhance their visualization. c) RDF for crystalline SnO slab (P4/nmm), the island from a), and the interface between the island from b) and the underlying slab.

Even though the obtained model (Figure 5) exhibits very high accuracy (95.7%), a physical interpretation of artificial neutral networks is not feasible. Therefore, we used the same dataset (Figure S5, Supporting Information) and applied a simpler 5D linear regression model (Figure S6, Supporting Information). This regression model is multidimensional as it is spanned by multiple features, whereby each *x* – *y* dependence was treated as linear. It has an accuracy of 88.9%, but it evidently shows that the average bond length within a cluster and its neighboring surface atoms (feature *x*1) and the number of the nearest neighbors within a cluster (feature *x*3) are the key physical parameters governing surface adsorption. It seems that the average electronegativity (feature *x*4) is not rendered significant by the model even though it is intuitive. This requires more testing in the future (e.g., other oxides or other ceramic systems). Nevertheless, a slight loss in precision of the regression model gives rise to easier transferability to DFT modeling, carried out in this study. Ten atoms were added onto a surface at a time to construct a cluster based on machine learning, which then underwent structural relaxation at 0 K. In total, 100 atoms with the Sn:O ratio of 1:1 were added. This is an enormous speedup in DFT modeling, which is always constrained in terms of length and time scales compared with classical molecular dynamics (e.g., growth of TiN was carried out using 25 000 steps per atom^[^ ^33^ ^]^). During this accelerated DFT modeling of SnO, it was assumed that surface diffusion is limited at room temperature so that adatoms cannot overcome the so‐called Ehrlich‐Schwoebel barrier, which is an additional energy barrier that adatoms encounter when they attempt to move from one atomic layer to another, particularly when descending or ascending a step edge.^[^ ^34^, ^35^ ^]^ If this hypothesis is shown to be correct (the experimental and modeling observations are consistent), the kinetic limitation is an important mechanism for the microstructure evolution of Sn─O thin films explored experimentally. The resulting Sn─O microstructure based on this accelerated DFT strategy is shown in **Figure** **6**. A key observation is that a SnO island forms on Sn (001) rather than a 2D layer (Figure 6A). This constitutes an initial stage of the dendrite formation. This configuration was thermalized at 300 K (growth temperature) for 10 000 steps. As its 3D nature is preserved, it clearly supports the hypothesis on kinetically limited surface diffusion. Furthermore, it can be speculated that another SnO island can form on the side of the existing one giving rise to branching, which is a fingerprint of dendritic microstructure. However, this SnO island (Figure 6A) seems to be amorphous, unlike the crystalline grains observed experimentally (Figure 3 and Figure S3, Supporting Information). If a seed layer is formed, it may promote crystallization. This hypothesis was tested by placing the amorphous SnO island onto crystalline SnO(001) and thermalizing it at 300 K for 10 000 steps. The obtained configuration is shown in Figure 6B. There are rows of Sn atoms ordering in the interfacial region, while O atoms tend to diffuse out of the interfacial region, which is consistent with the stacking sequence in crystalline SnO. Moreover, surface roughness decreases when Sn (Figure 6A) is replaced with the SnO seed layer (Figure 6B). Radial distribution functions (RDFs) were calculated for the amorphous SnO island, its thermalized counterpart on a seed layer, and the crystalline SnO seed layer (see Figure 6C). The amorphous island shows a continuous RDF above 2 Å, as expected, and exhibits a pre‐peak at 1.5 Å, which could be associated with short Sn─O bonds that are not present in the crystalline SnO counterpart. The SnO seed layer can be characterized by the typical appearance of RDFs in the case of crystalline substances, namely discrete and well‐defined peaks led by a high‐intensity peak at 2.3 Å. When the amorphous SnO island is placed on a crystalline SnO seed layer, important changes can be noticed. Clearly, the seed layer induces crystallization, which can be observed by the diminishing pre‐peak at 1.5 Å, the formation of a pronounced RDF shoulder at 2.3 Å, and the lack of the continuous background in the range of 2.5 Å. It is expected that more time is required to fully crystallize the SnO island, but the tendency is evidently present. Hence, our model rationalizes the existence of crystalline SnO islands with a tendency to form dendrites on Sn surfaces, as observed experimentally. It can be speculated that amorphous SnO exhibits different physical properties than crystalline SnO. For instance, reduced thermal conductivity is likely to occur. Other properties, e.g., optical, may also be drastically different. Our model can capture these structural modifications, which could be of relevance for applications.

## 3 Discussion

Modern technology often encounters setbacks due to the inadequacy of materials used. To overcome these challenges, a deeper understanding of the physics of novel materials is essential. Thin films, which are integral to a wide range of modern applications such as semiconductors, solar cells, and sensors, still require comprehensive studies in terms of their nucleation and growth processes. Traditional approaches, such as the structure zone diagram,^[^ ^2^ ^]^ are becoming outdated and should be replaced with knowledge‐based models that provide more accurate and predictive insights. In the case of Sn─O thin films covering the overlooked Sn‐rich corner of the corresponding phase diagram,^[^ ^29^ ^]^ the structure zone diagram does not capture the observed microstructure. A dendritic microstructure was observed (see Figure 3) rather than columnar. On the other hand, SnO_2_ thin films (Sn‐poor compositions) tend to satisfy the structure zone diagram.^[^ ^22^ ^]^ Dendrites can appear in samples grown from a liquid phase (e.g., TiO_2_ using hydrothermal process^[^ ^36^ ^]^), but are very unusual in gas phase synthesis.

To explain the unexpected dendritic microstructure of Sn_0.6_O_0.4_ thin films, DFT in tandem with machine learning was employed to construct a model, generically outlined in Figure 1. The synergy of DFT and AI provides a huge computational speedup (in the order of 10^7^ steps were saved). Our model contributes to the fundamental understanding of Sn─O thin film growth and underlying physics. Non‐equilibrium nanostructures, such as those explored in the current study, may address many shortcomings of Sn─O by combining self‐excluding physical properties (e.g., a combination of high electrical conductivity induced by Sn and low thermal conductivity promoted by the presence of SnO and resulting interfaces). The key scientific issue is their formation, but the available structure zone diagram does not provide answers. There are other attempts to address shortcomings of the structure zone diagram,^[^ ^2^ ^]^ such as the use of variational autoencoders and generative adversarial neural networks applied on process‐composition‐microstructure relations for predicting surface images of Cr─Al─O─N thin films.^[^ ^37^ ^]^ Other authors included the prediction of residual stresses in nitride thin films based on artificial neural networks.^[^ ^38^ ^]^ However, these approaches do not explicitly comprise atomic‐level descriptions, which may lead to a lack of physical connection to the smallest scale. Thus, we preserve the atomic scale in our model, which also allows for the analysis of electronic structure (DFT is the electronic structure method). The chosen Sn─O system seems to be kinetically limited, which promotes SnO island formation instead of a 2D layer. Another SnO island might form at the side of the existing one, which may induce branching, a sign of dendritic microstructure. Such amorphous islands on a SnO(001) seed flatten out and tend to crystallize. It can be suggested that Sn promotes dendritic growth (branching in Figure 3), while SnO imposes 2D microstructure (elongated features in Figure 3). This competitive growth scenario can be used to control the microstructure evolution by selecting a desired O/Sn ratio, a notion to be explored experimentally in future work. Furthermore, interface analysis would be beneficial to assess the applicability of the current machine learning model, but due to small and overlapping grains, such information is not accessible by TEM. Other techniques, such as tomography, may support additional analysis in future experiments. In addition, it can be envisioned that our model can be used to design novel Sn─O systems. For instance, Sn─O with microstructures containing both amorphous and crystalline regions may display a complex and fascinating interplay between order and disorder. This dual‐phase architecture may significantly influence their mechanical, thermal, optical, and transport properties.

Besides explaining the microstructure evolution of Sn_0.6_O_0.4_ thin films, our model may also be beneficial for related systems. This does not only include other Sn‐rich compositions but also core‐shell nanoparticles, which are advanced systems composed of a central core and a surrounding shell made of different substances, such as metallic Sn as a core and a SnO shell.^[^ ^26^ ^]^ This unique architecture allows for the combination of diverse properties, tailoring their functionality for various applications. The core often provides fundamental attributes such as key physical properties of interest, while the shell enhances stability (e.g., oxidation resistance). For instance, in batteries and fuel cells, core‐shell nanoparticles may enhance charge storage capacity and conductivity. Clearly, microstructure predictions are essential for many high‐tech applications. It remains to be seen if the proposed model (see Figure 1) is applicable to other systems, determining its generic and general use. More examples (diverse systems) should be considered, and additional AI features could be studied, e.g., activation energy. The Ehrlich‐Schwoebel barrier, closely related to the activation energy, is clearly central in describing surface diffusion on thin film surfaces, but our model is accelerated by considering clusters rather than single atoms, which may be explored in the future. More features, such as atomic radius, melting point, density, surface energy, oxidation state, electron affinity, ionization energy, enthalpy, bonding type, packing factor, defects, and preferred orientation, may also be helpful to improve the model or make it more general, but such a vast expansion of feature space would require substantial additional work. Replacing the structure zone diagram with AI models is still not fully possible, but this work provides relevant steps toward that end.

## 4 Conclusion

The formation of Sn and SnO nm‐sized crystallites leads to a dendritic thin film microstructure that diverges from the traditional structure zone diagram. A generic model for microstructure evolution, incorporating DFT and AI, has been proposed to explain this ambiguity. The model implies that the average bond length (within a cluster and its neighboring surface atoms) and the number of the nearest neighbors within a cluster are the key physical parameters governing surface adsorption. This was used to accelerate DFT aiming to uncover growth‐related physics. The growth of Sn_0.6_O_0.4_ thin films is suggested to be kinetically limited by Ehrlich‐Schwoebel potential barriers at step edges, giving rise to distinct SnO islands on Sn(001). This kinetic roughening is proposed to be the initial stage of dendritic microstructure evolution. The formation of another SnO island adjacent to the existing one likely leads to branching. This comprehensive approach provides valuable insights into the complex processes governing the microstructure evolution of overlooked Sn─O compositions relevant to many advanced applications, such as thermoelectric devices and batteries. By identifying the key parameters controlling early‐stage growth and introducing a predictive, AI‐accelerated framework, this work significantly advances the modeling of thin film growth beyond conventional paradigms.

## 5 Experimental Section

### Thin Film Growth

Sn─O thin film samples were synthesized in a vacuum system by employing reactive direct current (DC) magnetron sputtering. Si substrates were used in these deposition experiments without intentional heating, with the substrate (growth) temperature estimated to be ≈305 K. To warrant homogeneity across the Si surface, the substrates were rotated at 20 rpm. An elemental Sn target (99.95% purity, 50.8 mm in diameter, power density 5.8 W cm^−2^) was positioned 12.5 cm from the substrate and inclined at 40° to the substrate normal. Each specimen was synthesized over a 30‐minute period. The base pressure was ≈3 × 10^−6^ Pa and the Sn─O thin film samples were grown in an Ar/O_2_ atmosphere at a total working pressure of 0.5 Pa. The O_2_ partial pressure was adjusted to obtain mixtures of Sn and SnO, with values of 0.03, 0.04, and 0.05 Pa being tested (balanced with Ar to maintain a constant working pressure of 0.5 Pa in all depositions). The sample grown at the highest O_2_ partial pressure was analyzed in detail and presented throughout this work.

### Thin Film Characterization

The composition of the DC magnetron sputtered Sn─O samples was revealed by EDX, operated in a Zeiss EVO LS10 SEM instrument, equipped with a LaB_6_ filament. Energy calibration of the EDX detector was performed using a Co reference sample. Three EDX measurements were conducted at an accelerating voltage of 15 kV to estimate the error bars. The structure of these Sn─O thin films was analyzed employing WAXS in an XEUSS 3.0 instrument (Cu source with a wavelength of 1.5406 Å), with the detector positioned 73 mm from the sample. The x‐ray beam grazed the sample surface at an angle of 2.5°. Additionally, high‐resolution TEM analysis was performed using a Talos F200X microscope at an accelerating voltage of 200 kV. Carbon was used in the pre‐deposition process to avoid the bombardment of the film surface by the ion beam. The Pt was deposited to further protect the samples during the preparation of cross‐sections via focused ion beam (FIB) milling, which was carried out using a Thermo Fisher Scientific Helios G4 CX platform. EDX and SAED were also performed in conjunction with TEM.

### Density Functional Theory

DFT^[^ ^39^ ^]^ modeling served a twofold purpose (see Figure 1): i) calculation of adsorption energy at 0 K for Sn─O clusters on SnO(001) serving as input for machine learning and ii) description of microstructure evolution (accelerated DFT‐based on machine learning). For both purposes the OpenMX code,^[^ ^40^ ^]^ was employed, featuring a linear combination of localized pseudoatomic orbitals^[^ ^41^ ^]^ and the generalized gradient approximation within the Perdew, Burke, and Ernzerhof parametrization.^[^ ^42^ ^]^ The basis set was designated as Sn7.0‐s2p2d3f1 and O7.0‐s2p2d1, where the chemical symbol was followed by the cut‐off radius (expressed in the Bohr atomic units) and the corresponding orbitals. An energy cut‐off of 150 Ry (2041 eV) was selected to achieve a total energy precision of 10^−6^ Ry (13.6 µeV) with a real‐space grid of 112 × 112 × 200 (case i) and 147 × 147 × 252 (case ii). Spin polarization was not considered. A slab model was employed with a vacuum thickness of 15 Å. The structure was visualized using VESTA^[^ ^43^ ^]^ and the RDF was obtained using OVITO.^[^ ^44^ ^]^ For the calculation of adsorption energy at 0 K (case i), a SnO slab with 192 atoms was created from bulk SnO (P4/nmm), as shown in Figure S4, Supporting Information. Atoms were randomly placed 2 Å above the surface, forming clusters. Units containing 1 to 7 Sn and/or O atoms were probed. Structural relaxations at 0 K were then followed by the calculation of the adsorption energy, which was obtained by subtracting the energy of the slab (SnO(001) with a cluster) from that of the constituent counterparts. For the description of microstructure evolution (case ii), two slabs were used: Sn(001) and SnO(001). Ten atoms, with a Sn:O ratio of 1:1, were randomly placed 2 Å above the Sn slab, containing 144 atoms. These atoms were clustered according to the machine learning outcome to produce the most stable unit, which accelerated DFT (otherwise molecular dynamics should be performed with at least 1000 steps per atom). In total 100 atoms were added by sequentially introducing sets of 10 atoms each time, which constituted the final configuration of 244 atoms. After the addition of each set, the whole configuration was relaxed at 0 K. Finally, the configuration was thermalized (equilibrated) at 300 K using DFT‐based molecular dynamics (10 000 steps, canonical ensemble, and velocity scaling). The relaxed atomic coordinates were available in Supporting Information. The resulting SnO island with 100 atoms was then placed on SnO(001), containing 128 atoms, and thermalized in the same fashion. Both configurations, Sn(001) and SnO(001) based, were relaxed at 0 K after the respective thermalizations.

### Machine Learning

The DFT dataset with 93 adsorption energies (see Supporting Information) served as input for the artificial neural network.^[^ ^45^ ^]^ Hence, the same clusters were considered in the machine learning model as in DFT (see Figure 4). The corresponding Python script was available in Supporting Information. The DFT dataset was preprocessed to ensure consistency and accuracy, which included normalizing feature scales. Next, applicable features were identified and selected: *x*1 (average bond length within a cluster and its neighboring surface atoms), *x*2 (number of the nearest neighbors with the SnO(001) surface), *x*3 (number of the nearest neighbors within a cluster), and *x*4 (average electronegativity^[^ ^32^ ^]^ for *x*2). The architecture of the artificial neural network was established, including the number of input features, hidden layers, and neurons per layer to maximize the accuracy of the model.^[^ ^45^ ^]^ The rectified linear unit was selected for the activation function^[^ ^45^ ^]^ and regularization of 0.001 was adopted to prevent overfitting. The learning rate was constant. The DFT dataset was randomly split into training and validation sets, and the artificial neural network was trained on the training set (80% of the data) while monitoring performance on the validation set to prevent overfitting. The Adam optimizer was selected for the optimization algorithm.^[^ ^45^ ^]^ The performance (accuracy) was evaluated using the *R* ^2^ coefficient of determination.

### Statistical Analysis

Sn─O thin films were grown in two separate batches. By comparing the composition and structure of the samples from both batches, reproducibility was confirmed. For the EDX measurements, each sample was analyzed three times at different surface locations, and the results were averaged to determine the composition. Error bars were calculated based on this method. Although no statistical analysis was performed for the WAXS and TEM measurements, the proper functioning of the corresponding instruments was verified prior to data collection. Machine learning with 93 data points from DFT included statistical tools, such as the coefficient of determination, as incorporated in the Python code (see Supporting Information).

## Conflict of Interest

The authors declare no conflict of interest.

## Supporting information



Supporting Information

## Data Availability

The data that support the findings of this study are available in the supplementary material of this article.
