# Supplementary material for: Kinetically Limited Growth of Dendritic Tin Oxide Thin Films: a Machine Learning Study beyond the Structure Zone Diagram
Source: Adv Sci (Weinh). 2025 May 29;12(32):e04627. doi: 10.1002/advs.202504627 (PMC12407291; doi:10.1002/advs.202504627)
Supplement: Supplementary file 1 — Supporting Information [file ADVS-12-e04627-s001.pdf]

ADVANCED SCIENCE
Open Access

Supporting Information

for *Adv. Sci.*, DOI 10.1002/advs.202504627

Kinetically Limited Growth of Dendritic Tin Oxide Thin Films: a Machine Learning Study beyond the Structure Zone Diagram

Denis Music, Xuelian Xiao, Rami Naser, Keke Chang*, Grzegorz Sadowski and Pär A. T. Olsson*

Supporting Information

Kinetically Limited Growth of Dendritic Tin Oxide Thin Films: A Machine Learning Study Beyond the Structure Zone Diagram

Denis Music, Xuelian Xiao, Rami Naser, Keke Chang,* Grzegorz Sadowski, and Pär A. T. Olsson*

The data presented in Supplementary Information provide additional experimental support (Figure S1, Figure S2, and Figure S3) and theoretical details (bare SnO(001) slab in Figure S4, visualized DFT dataset in Figure S5, linear regression model in Figure S6, the used Python script, DFT input dataset, and atomic coordinates for a SnO island on Sn(001)).

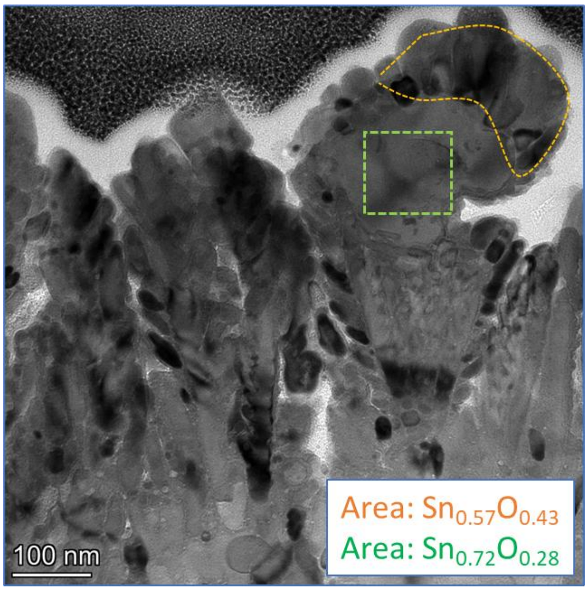

Figure S1. EDX data for a Sn-O thin film. The assessment was carried out in conjunction with the TEM analysis. Two regions were evaluated (marked in the figure).

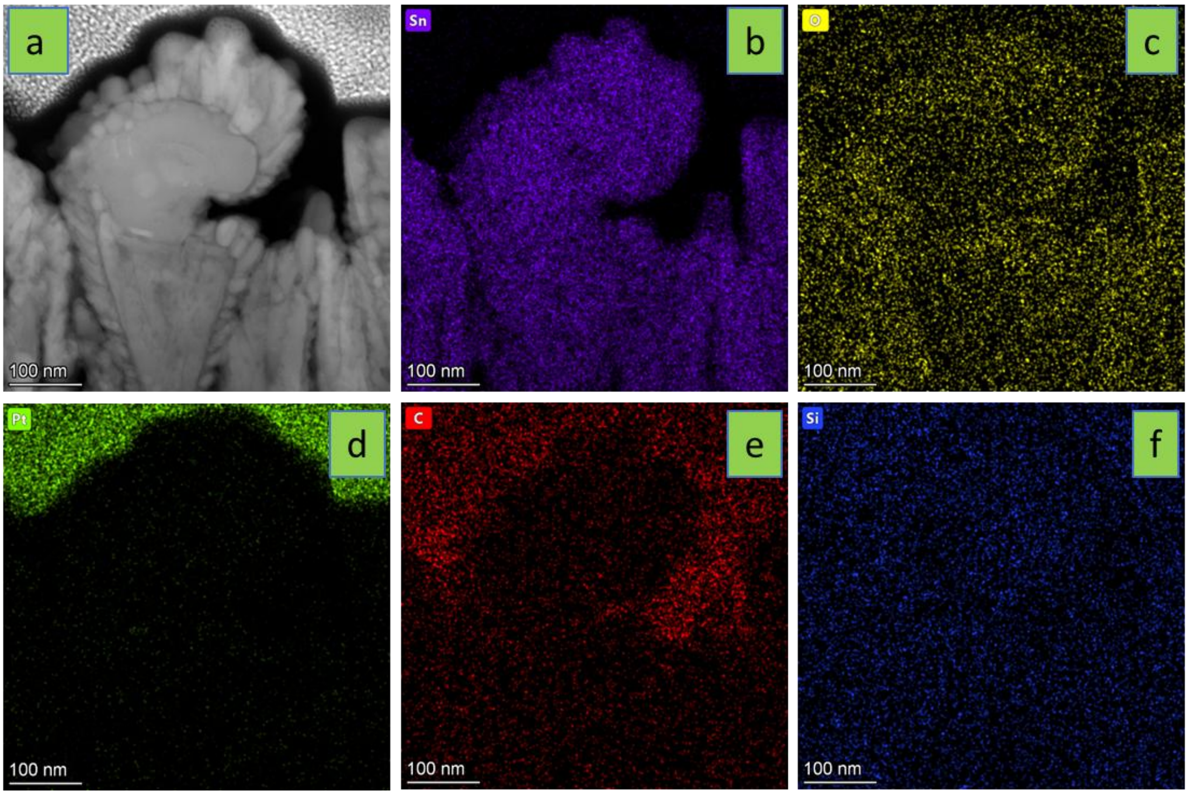

Figure S2. High-angle annular dark-field (HAADF) analysis. a) TEM image of the thin film analyzed. HAADF provides atomic number contrast: b) for Sn, c) for O, d) for Pt (protective layer during FIB milling), e) for C (protective layer), and f) for Si (substrate). Small fluctuations in the Sn and O content are present, being consistent with the EDX data in Figure S1.

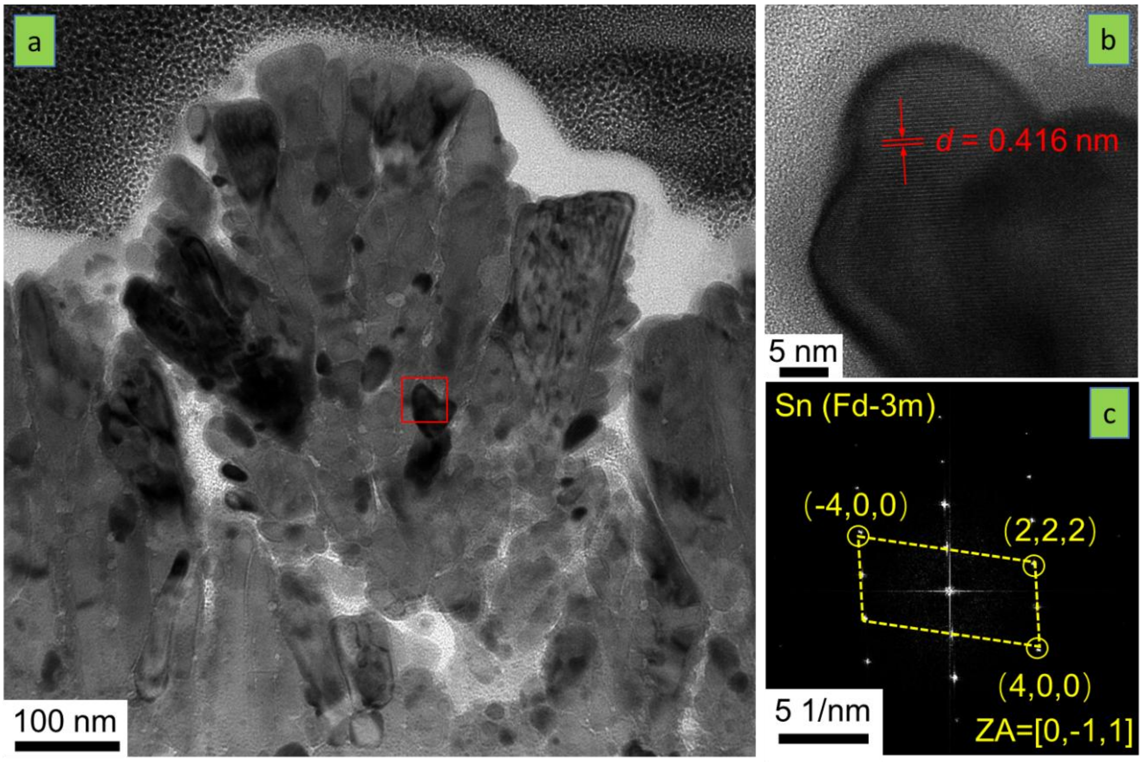

Figure S3. TEM analysis of $Sn_{0.6}O_{0.4}$. a) Bright-field image of the sample analyzed. The area marked by the square is where the high-resolution TEM assessment was carried out. b) High-resolution TEM of a grain exhibiting Sn structure (Fd-3m). The corresponding SEAD pattern is analyzed in image c).

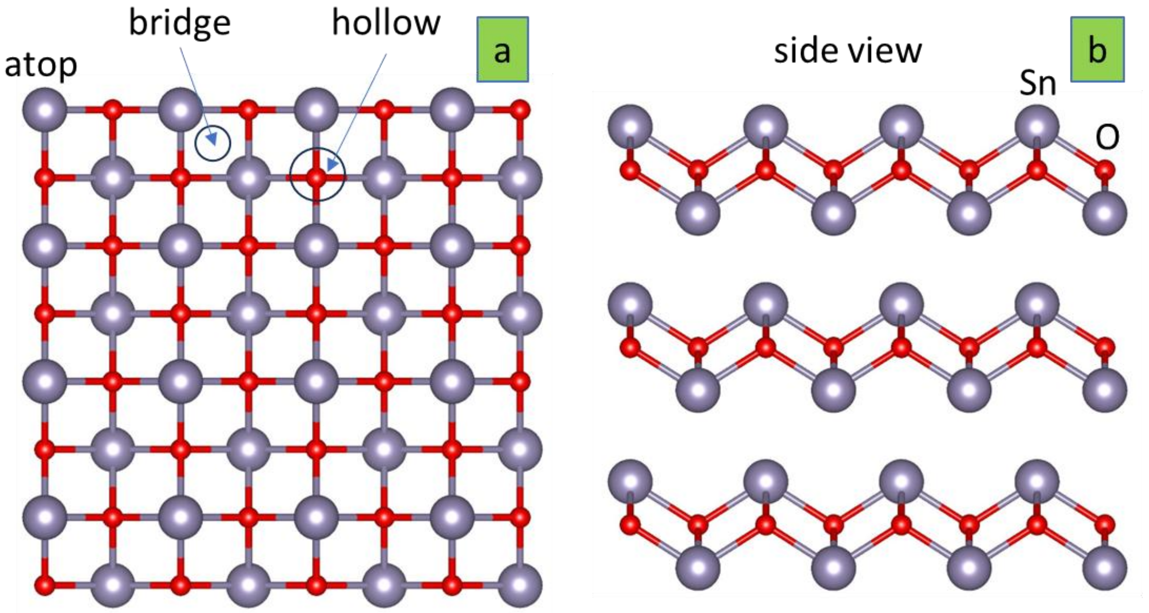

Figure S4. Possible SnO(001) surface sites. a) The top view is provided with atop, bridge, and hollow sites indicated. b) Side view of SnO(001) is given.

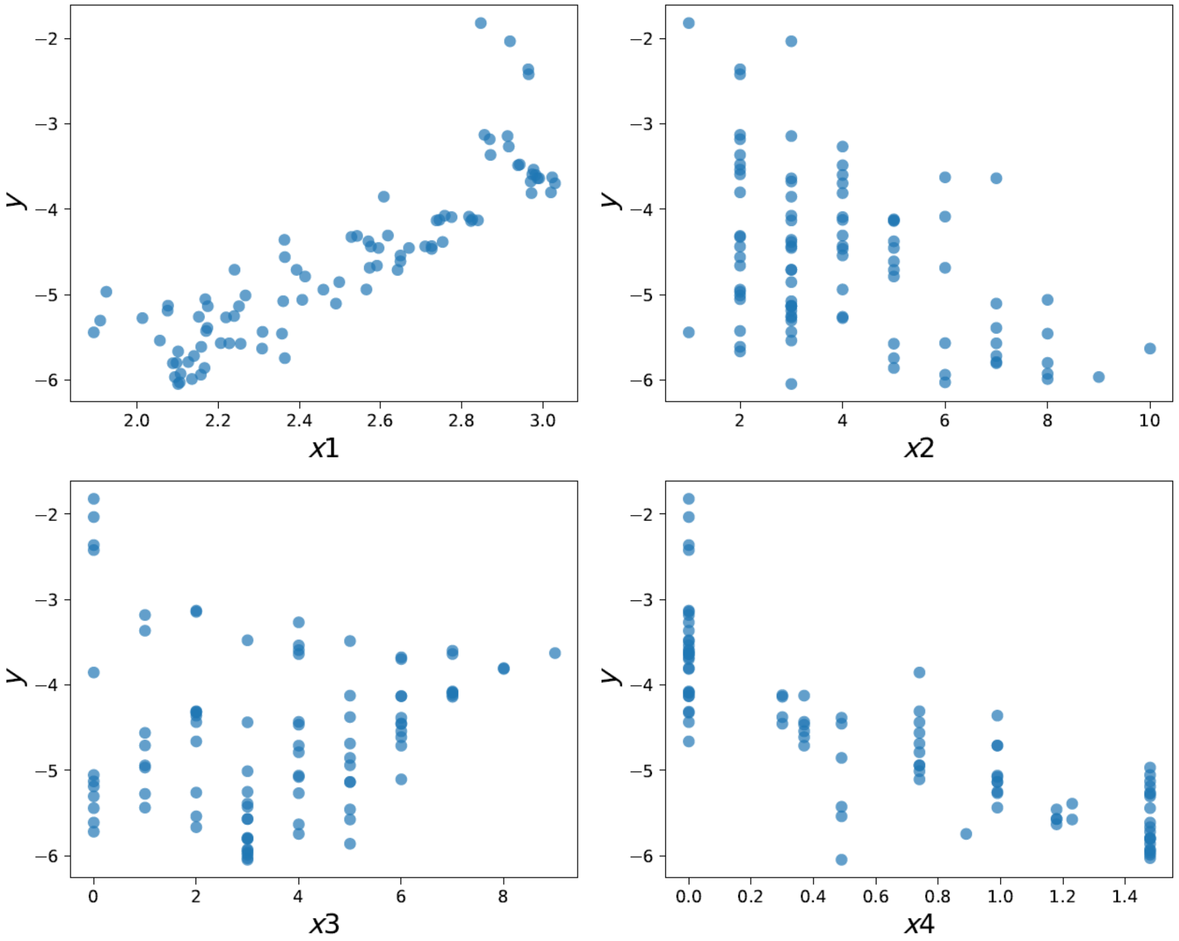

Figure S5. The raw DFT dataset. *y* is the calculated adsorption energy in eV/atom, *x*1 designates the average bond length within a cluster and its neighboring surface atoms in Å, *x*2 is the number of the nearest neighbors with the SnO surface, *x*3 describes the number of the nearest neighbors within a cluster, and *x*4 is the average electronegativity for *x*2.

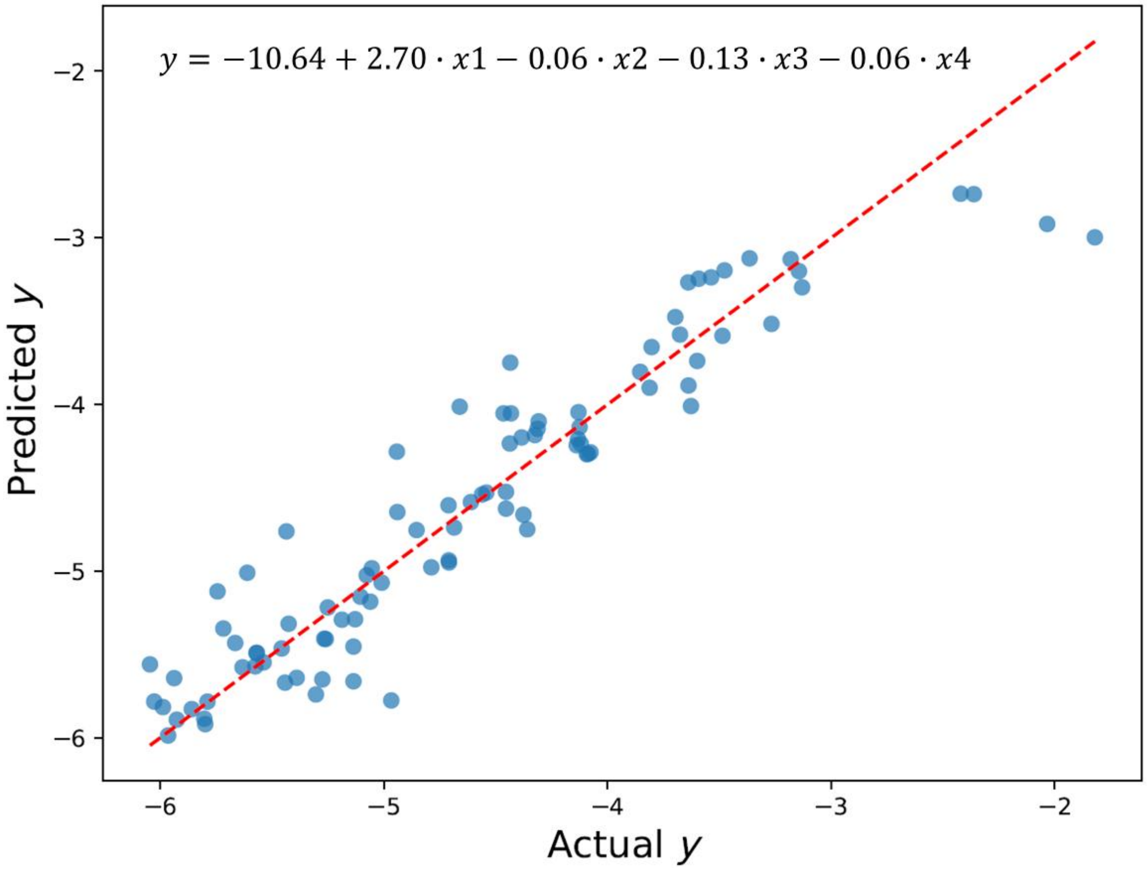

Figure S6. Linear regression model. *y* is the calculated adsorption energy in eV/atom. The accuracy of 88.9% was achieved. The inset shows the mathematical expression obtained from the linear regression model.

The Python script for the artificial neural network used in this study ("data.txt" is provided below in which the columns are: *y* (adsorption energy in eV/atom), *x*1 (average bond length within a cluster and its neighboring surface atoms in Å), *x*2 (number of the nearest neighbors with the SnO surface), *x*3 (number of the nearest neighbors within a cluster), and *x*4 (average electronegativity for *x*2)).

```
import numpy as np
from sklearn.metrics import mean_squared_error, r2_score
from sklearn.neural_network import MLPRegressor
from sklearn.model_selection import GridSearchCV, train_test_split
from sklearn.preprocessing import StandardScaler
data = np.loadtxt('data.txt', delimiter='\t', skiprows=1)
y = data[:, 0]
X = data[:, 1:]
X_train, X_test, y_train, y_test = train_test_split(X, y, test_size=0.2, random_state=42)
scaler_X = StandardScaler()
scaler_y = StandardScaler()
X_train_scaled = scaler_X.fit_transform(X_train)
y_train_scaled = scaler_y.fit_transform(y_train.reshape(-1, 1)).flatten()
X_test_scaled = scaler_X.transform(X_test)
y_test_scaled = scaler_y.transform(y_test.reshape(-1, 1)).flatten()
parameter_space = {
    'hidden_layer_sizes': [(64, 32), (100, 50), (50, 25, 10)],
    'activation': ['relu', 'tanh'],
    'solver': ['adam', 'lbfgs'],
    'alpha': [0.0001, 0.001, 0.01],
    'learning_rate': ['constant', 'adaptive'],
    'max_iter': [3000]
}
model = MLPRegressor(random_state=1)
clf = GridSearchCV(model, parameter_space, n_jobs=1, cv=5)
clf.fit(X_train_scaled, y_train_scaled)
best_model = clf.best_estimator_
best_model.fit(X_train_scaled, y_train_scaled)
y_pred_scaled = best_model.predict(X_test_scaled)
y_pred = scaler_y.inverse_transform(y_pred_scaled.reshape(-1, 1)).flatten()
y_train_pred_scaled = best_model.predict(X_train_scaled)
y_train_pred = scaler_y.inverse_transform(y_train_pred_scaled.reshape(-1, 1)).flatten()
y_test_pred_scaled = best_model.predict(X_test_scaled)
y_test_pred = scaler_y.inverse_transform(y_test_pred_scaled.reshape(-1, 1)).flatten()
mse = mean_squared_error(y_test, y_pred)
r2 = r2_score(y_test, y_pred)
print(f"Best Parameters: {clf.best_params_}")
print(f"Mean Squared Error: {mse}")
print(f"R^2 Score: {r2}")
```

y	x1	x2	x3	x4
-2.421	2.965	2	0	0.00
-1.821	2.847	1	0	0.00
-2.362	2.964	2	0	0.00
-3.365	2.871	2	1	0.00
-3.181	2.869	2	1	0.00
-2.034	2.919	3	0	0.00
-3.130	2.856	2	2	0.00
-3.144	2.913	3	2	0.00
-3.477	2.943	2	3	0.00
-3.639	2.987	3	4	0.00
-3.537	2.977	2	4	0.00
-3.592	2.974	2	4	0.00
-3.676	2.970	3	6	0.00
-3.267	2.916	4	4	0.00
-3.803	3.020	2	8	0.00
-3.697	3.030	4	6	0.00
-3.486	2.939	4	5	0.00
-3.599	2.982	4	7	0.00
-3.812	2.972	4	8	0.00
-3.627	3.023	6	9	0.00
-3.638	2.991	7	7	0.00
-5.611	2.158	2	0	1.48
-5.442	1.893	1	0	1.48
-5.054	2.168	2	0	1.48
-4.967	1.924	2	1	1.48
-5.188	2.075	3	0	1.48
-5.129	2.076	3	0	1.48
-5.718	2.140	7	0	1.48
-5.304	1.909	3	0	1.48
-5.275	2.013	4	1	1.48
-4.561	2.364	2	1	0.74
-4.942	2.459	2	1	0.74
-3.854	2.608	3	0	0.74
-4.435	2.710	3	2	0.00
-4.325	2.528	2	2	0.00
-4.661	2.591	2	2	0.00
-5.436	2.309	3	1	0.99
-4.709	2.240	3	1	0.99
-5.665	2.101	2	2	1.48
-4.465	2.726	4	4	0.37
-4.432	2.726	4	4	0.37
-4.313	2.542	2	2	0.00
-5.010	2.267	2	3	0.74
-4.359	2.363	3	2	0.99
-4.437	2.576	2	3	0.74
-6.046	2.101	3	3	0.49
-5.538	2.056	3	2	0.49
-5.426	2.170	2	3	0.49
-4.132	2.738	3	6	0.00
-4.125	2.745	4	5	0.37
-4.384	2.753	3	6	0.49
-4.710	2.393	3	4	0.99
-4.308	2.618	4	2	0.74
-4.940	2.565	4	5	0.74
-5.744	2.364	5	4	0.89
-5.251	2.239	3	3	0.99
-5.077	2.360	3	4	0.99
-5.938	2.157	6	3	1.48
-5.260	2.152	4	2	1.48
-6.027	2.105	6	3	1.48
-4.076	2.758	3	7	0.00
-4.130	2.840	5	6	0.00
-4.138	2.823	5	7	0.30
-4.454	2.595	3	6	0.49
-4.542	2.649	4	6	0.37
-4.686	2.573	6	5	0.74
-5.267	2.219	3	4	0.99
-5.136	2.251	3	5	0.99
-4.854	2.498	3	5	0.49
-5.570	2.227	7	3	1.18
-5.568	2.206	6	3	1.18
-5.859	2.166	5	5	1.48
-5.988	2.135	8	3	1.48
-5.789	2.126	7	3	1.48
-5.926	2.107	8	3	1.48
-4.093	2.775	4	7	0.00
-4.087	2.818	6	7	0.00
-4.119	2.826	5	7	0.30
-4.454	2.670	5	6	0.30
-4.376	2.570	5	5	0.30
-4.611	2.649	5	6	0.37
-5.105	2.490	7	6	0.74
-4.788	2.414	5	4	0.74
-4.711	2.642	5	6	0.37
-5.457	2.357	8	5	1.18
-5.136	2.174	3	5	0.99
-5.061	2.407	8	4	0.99
-5.632	2.308	10	4	1.18
-5.390	2.173	7	3	1.23
-5.575	2.255	5	5	1.23
-5.965	2.093	9	3	1.48
-5.799	2.097	8	3	1.48
-5.803	2.088	7	3	1.48

The Cartesian coordinates for the Sn/SnO slab model used in DFT (see Figure 6A). SnO is an island formed on Sn(001).

Sn	0.97497	0.98373	8.81847
Sn	0.99901	1.68757	15.27797
Sn	1.01810	7.75085	8.87343
Sn	1.05181	7.31090	16.25311
Sn	0.88816	14.07560	8.96759
Sn	1.77905	15.00960	15.46237
Sn	7.72373	0.71732	9.20491
Sn	7.34125	0.85130	15.96334
Sn	7.71816	7.52186	9.24825
Sn	7.66864	7.91556	16.09804
Sn	7.71723	14.29819	8.85598
Sn	6.92740	14.57461	15.67482
Sn	14.25320	1.00258	9.00025
Sn	13.97829	0.44099	16.38372
Sn	14.36730	7.39502	9.16723
Sn	15.59873	8.48078	15.64000
Sn	14.32224	14.19630	8.98664
Sn	14.16441	14.75512	15.54475
Sn	4.42563	4.12417	9.03316
Sn	3.48413	3.43045	15.62638
Sn	4.31172	10.84756	9.04161
Sn	4.80178	11.31977	14.94470
Sn	4.15412	17.58811	8.88557
Sn	5.39194	18.58500	16.05896
Sn	10.98677	4.15370	9.57142
Sn	10.83084	4.54317	15.94724
Sn	11.02909	10.83253	9.14880
Sn	11.69403	12.42042	15.12284
Sn	11.10514	17.62202	9.42987
Sn	12.26711	18.18430	15.44434
Sn	17.68160	4.09401	9.24038
Sn	16.95214	5.91596	17.32543
Sn	17.57018	10.73471	9.31414
Sn	17.89634	11.34200	15.54549
Sn	17.77065	17.72475	9.00210
Sn	17.18958	16.22033	15.84039
Sn	4.30239	0.88143	5.51279
Sn	4.20942	0.70163	12.29138
Sn	4.41408	7.68365	5.60025
Sn	4.17619	7.38284	12.42067
Sn	3.84365	14.49935	5.60609
Sn	4.28089	14.36744	11.94780
Sn	11.00488	0.87185	5.89345
Sn	11.12802	0.92814	12.75848
Sn	11.21990	7.28073	5.87558
Sn	11.10908	7.64102	12.80044
Sn	10.98308	14.23117	5.72181
Sn	11.00347	14.19420	12.28162
Sn	17.80184	0.54500	5.46964
Sn	17.60823	0.86504	12.54389
Sn	17.64752	7.37921	5.79145
Sn	17.88269	7.44422	12.58496
Sn	17.53572	14.08594	5.81140
Sn	17.65612	14.20103	12.29866
Sn	0.88891	4.22168	5.63244
Sn	0.87189	4.14084	12.13646
Sn	0.72368	11.09153	5.62445
Sn	1.00005	10.84619	12.45168
Sn	0.21432	18.12935	5.55675
Sn	0.85370	17.71592	12.27945
Sn	7.85549	4.41296	5.65497
Sn	7.55693	4.14100	12.56184
Sn	7.58879	10.94218	5.71826
Sn	7.69584	10.96451	12.25038
Sn	7.69615	17.73979	5.71267
Sn	7.45633	17.38334	12.66030
Sn	14.21649	4.26082	5.73136
Sn	14.45343	4.21042	12.82310
Sn	14.18616	10.95267	5.71804
Sn	14.24234	10.72414	12.57144
Sn	14.01322	17.76240	5.74728
Sn	14.79181	17.72983	12.27394
Sn	4.83472	4.66353	5.45335
Sn	2.64852	2.46524	10.61503
Sn	1.58885	8.36257	5.01786
Sn	2.67296	9.21660	10.75021
Sn	1.09476	15.45596	4.76511
Sn	2.40532	15.99387	10.50788
Sn	8.38321	1.66479	4.91668
Sn	9.26704	2.35658	11.03914
Sn	8.64422	8.34278	4.95155
Sn	9.50367	9.16386	10.89368
Sn	8.74404	15.43045	4.23612
Sn	9.19161	15.93858	10.77721
Sn	15.07549	1.53538	4.96740
Sn	15.87341	2.49963	10.89987
Sn	15.08612	8.34994	4.80142
Sn	15.99636	9.00150	10.99129
Sn	14.92614	15.10385	5.02205
Sn	16.14373	15.88215	10.51116
Sn	6.62506	6.50715	3.68450
Sn	5.98072	5.79032	10.79043
Sn	6.51797	13.59138	4.92723
Sn	6.05797	12.60139	10.49667
Sn	6.46388	19.69668	3.85778
Sn	5.93741	19.00646	10.72300
Sn	13.12820	6.27652	3.82811
Sn	12.75460	5.90171	11.09356

Sn	13.03660	13.07522	3.95425
Sn	12.76573	12.46468	10.76079
Sn	12.88886	19.78509	3.89550
Sn	12.84491	19.40499	10.92582
Sn	19.71465	6.32784	3.95419
Sn	19.42155	6.02171	10.53309
Sn	19.46171	13.09260	3.80757
Sn	19.31822	12.45178	10.76194
Sn	2.96786	2.99796	3.92920
Sn	19.32336	19.50189	10.65050
Sn	6.06908	2.31265	7.38254
Sn	5.75847	2.35423	14.03640
Sn	6.08296	9.17482	7.49369
Sn	6.17406	9.01980	13.78120
Sn	5.92536	15.96463	7.22312
Sn	5.10756	16.22859	14.07611
Sn	12.47586	2.76137	7.51907
Sn	12.78567	2.51082	14.59768
Sn	12.69599	9.15630	7.51336
Sn	12.62367	9.43585	14.69705
Sn	12.48704	15.95458	7.51361
Sn	12.75625	16.12847	13.46813
Sn	19.12489	2.51717	7.23599
Sn	18.86367	3.09344	13.90346
Sn	19.12809	9.33707	7.34442
Sn	19.56683	9.14954	14.31831
Sn	19.25739	15.84779	7.34224
Sn	19.41539	15.84941	13.89810
Sn	2.61089	5.77440	7.41393
Sn	2.28957	5.77601	14.03351
Sn	2.65521	12.44825	7.31502
Sn	2.66250	12.84349	13.73581
Sn	2.51415	19.21446	7.10528
Sn	2.41755	19.33401	14.14815
Sn	9.47281	5.79314	7.70025
Sn	9.06897	6.05174	14.15039
Sn	9.43181	12.45234	7.38402
Sn	8.69392	13.25093	13.78076
Sn	9.48258	19.09701	7.57814
Sn	9.08641	19.38079	14.19760
Sn	16.02250	5.65543	7.50865
Sn	-3.20520	5.34130	14.31718
Sn	16.00083	12.31421	7.51633
Sn	15.83296	12.67692	14.02987
Sn	15.91854	19.25059	7.36058
Sn	15.99528	19.34437	14.47697
O	14.16256	16.40792	16.92404
Sn	3.35272	14.92566	18.54032
Sn	8.02203	14.80651	18.46580
O	8.50287	11.34234	18.42179
O	6.35671	12.41973	15.95958
Sn	8.02700	11.02114	16.38240
O	12.77263	16.91553	17.15619
Sn	9.53645	6.25001	18.80244
O	7.15307	17.46579	17.21650
Sn	9.25367	18.09630	17.80100
Sn	15.88077	11.01277	17.70593
O	3.62431	16.99872	17.84325
Sn	6.61432	1.30667	19.48902
O	2.64049	18.66190	20.46079
Sn	10.66981	11.21425	18.68735
O	18.46392	15.25456	17.46352
Sn	7.47334	3.86647	16.91176
O	9.58406	12.29260	15.56755
Sn	4.78045	6.63625	16.25953
O	3.09267	8.05810	16.49786
Sn	15.31482	8.36070	19.12225
O	12.48127	13.79138	16.60493
Sn	19.54380	13.61092	16.64185
O	11.32514	6.08325	17.37091
Sn	1.61627	2.62866	17.99382
O	10.57806	11.02851	20.84007
Sn	18.11429	8.76132	17.86217
O	9.51450	3.99559	17.49002
Sn	1.97392	17.88967	16.75357
O	4.96764	5.85694	18.23662
O	9.76447	15.98640	18.00851
Sn	12.37613	7.93125	17.70440
O	1.30105	14.88875	19.10303
Sn	1.31691	19.70442	19.11030
O	15.06253	7.09084	17.30763
Sn	4.58348	3.97764	19.25432
O	17.21666	18.29173	15.78843
O	6.73462	3.44501	18.90521
Sn	4.04011	0.84962	17.73048
O	10.14337	18.29991	15.79151
Sn	11.78879	15.01125	18.42628
O	19.69294	12.30866	18.39179
Sn	4.59794	18.05741	19.47497
Sn	5.64213	7.39919	19.58035
O	10.60881	13.27043	19.20896
Sn	18.18921	12.26448	19.91427
O	6.76780	16.66248	18.43890
O	10.11595	5.75110	21.11940
O	5.63691	8.47736	17.54119
Sn	10.43145	13.15468	21.44664
Sn	19.43464	16.07585	19.34708
Sn	7.15486	4.37692	21.80300
O	8.82180	6.30181	21.66738
Sn	11.82361	7.25410	21.24061
O	18.91458	14.21906	20.45441
Sn	4.02165	9.82539	17.29958

O	6.27647	6.24918	22.96614
O	3.99627	16.16475	20.53071
O	10.97293	7.91840	19.35568
Sn	0.63549	10.50577	17.70668
O	1.40153	10.38585	19.71153
O	3.02346	3.68501	22.08598
O	6.88309	6.14936	24.33321
Sn	5.84553	15.13463	21.38351
Sn	3.48114	10.12397	20.20445
O	0.86007	15.97680	21.67821
Sn	0.99583	13.93248	21.09454
Sn	2.53923	17.08794	21.95051
Sn	1.30943	3.94978	20.76280
O	4.58655	13.55003	21.84925
O	5.25589	5.28610	20.89990
Sn	9.40995	9.98979	22.22652
O	10.66726	8.40334	22.58608
Sn	7.03734	11.77280	20.02339
O	3.48944	7.97166	19.92364
Sn	4.84131	17.90114	23.97196
O	3.20500	9.12570	24.29839
Sn	4.44104	12.65890	23.70576
O	8.22032	12.97185	21.40175
Sn	1.70912	7.00886	20.58485
Sn	6.18966	9.30556	22.71432
O	5.37884	3.71955	23.60794
O	6.20847	11.38312	23.46352
Sn	5.69957	15.12737	25.33836
Sn	4.31165	1.97102	22.69968
O	4.42513	16.75688	25.61975
Sn	7.86441	12.78304	23.58679
Sn	4.18674	5.34016	22.72341
O	7.64768	10.00145	21.13058
O	5.65340	2.32135	21.13309
Sn	0.07025	9.56823	21.20927
O	5.92186	16.10411	23.34672
O	2.64815	6.69399	22.50708
O	9.59917	11.58490	23.45924
Sn	2.25275	7.30825	24.62178
Sn	5.64410	4.78428	25.52631
Sn	9.19016	7.03078	23.80420
O	8.13167	8.86968	23.56794
O	3.89663	5.78857	24.76182
Sn	2.39988	10.53506	23.04353
